# Comparing the effects of physical activity and cognitive training on cognitive performance, physical fitness, and mental health in 9- to 10-year-old children: a randomized clinical trial

**DOI:** 10.3389/fpsyg.2025.1555451

**Published:** 2025-06-26

**Authors:** Shanshan Wang, Jingwu Liu

**Affiliations:** Department of Physical Education, Changzhi University, Changzhi, China

**Keywords:** exercise, physical fitness, cognition, mental health, children

## Abstract

**Aim:**

This study aimed to compare the effects of physical activity (PA), cognitive training (CT), and their combination (PA+CT) on cognitive performance, physical fitness, and mental health in children aged 9–10 years using a randomized controlled trial (RCT).

**Methods:**

This RCT assigned 145 children (9.74 ± 0.43 years, 46% girls) into four groups: Con (no intervention), PA (aerobic exercises), CT (cognitive tasks), and PA+CT (combined PA and CT). All interventions were administered four times each week for 12 weeks, with 40-min sessions per intervention. The PA group underwent regular physical activity, the CT group received cognitive training, and the PA+CT group combined both activities. Key anthropometric measurements [including height, weight, and BMI body mass index (BMI)], physical fitness tests (including vital capacity, flexibility quality, speed quality, aerobic performance, and physical coordination), cognitive function assessments (including attention, reaction time, and spatial memory), and mental health evaluations (including anxiety and depression) were conducted before and after the intervention.

**Results:**

The results demonstrated no significant differences in body composition among the groups (*p* > 0.05). The results of physical fitness revealed that PA, CT, and PA+CT interventions can significantly improve physical fitness parameters in children (*p* < 0.05); although CT alone showed no significant impact (*p* > 0.05). The study found that all cognition and mental health parameters improved significantly in the PA, CT, and PA+CT groups than in the control group (*p* < 0.05), with the strongest effects in PA+CT.

**Conclusion:**

This study demonstrates that structured interventions administered four times each week can differentially improve physical fitness, cognition, and mental health outcomes in school-aged children. The synergistic effects observed in the combined PA+CT group underscore the value of integrating physical and cognitive training into school health programs.

## 1 Introduction

A significant increase in low physical fitness levels, as well as negative secular declines in physical fitness, particularly for cardiorespiratory endurance and muscle strength and power, in school-aged children, is a global public health concern, as is the low prevalence of adolescents meeting recommended levels of PA (Chen et al., 2020). Such deficits collectively contribute to multidimensional challenges in youth populations, encompassing physical fitness deterioration, cognitive impairments, and mental health vulnerabilities (Hadier et al., 2024). Consequently, developing effective strategies to enhance children’s overall health has emerged as a critical research focus at the intersection of public health and educational policy (Hadier et al., 2025).

Proponents of PA have long argued that school-affiliated sports are needed, suggesting that the time spent in PA benefits both physical and mental health, and might also contribute to academic performance (Donnelly et al., 2016). On the one hand, PA can improve physical fitness and academic achievement. Most of the evidence demonstrated that long-term exercise interventions effectively reduce adiposity (Lee, 2021), improve physical fitness in tasks such as the 20-m shuttle run, standing long jump, grip strength, 4 × 10 m shuttle run, and sit and reach (Zhang et al., 2023) and enhance academic achievement (Takehara et al., 2021). On the other hand, children’s exercise experiences help improve their cognition and mental health, both of which are vital for navigating challenges later in life. PA can lead to improvements in self-esteem (Liu et al., 2015) and attenuated depression symptomatology (Biddle and Asare, 2011). Emerging evidence suggests that exercise-induced cognitive enhancement is primarily due to cerebrovascular optimization, particularly through improved cerebral oxygenation mediated by augmented blood flow dynamics (Querido and Sheel, 2007). Hence, implementing well-designed human trials remains imperative to validate these mechanisms and expand clinical applications of cognitive enhancement strategies.

The World Health Organization (WHO) guidelines emphasize that children and adolescents should engage in 60 min of daily moderate-to-vigorous PA to improve their health outcomes (Baran et al., 2020). Current physical education paradigms incorporate multiple intervention modalities: conventional exercise programs (e.g., structured aerobic training) (Villa-González et al., 2023), technology-enhanced interventions (e.g., adaptive exergaming and game-based dual-task training) (McKay et al., 2019; Szturm et al., 2022), and cognitive-focused regimens (e.g., computerized attention training) (Xie et al., 2021). However, there is still a significant further study to explore and compare different exercise interventions for physical and mental health in children. This paucity of comparative effectiveness research highlights a critical knowledge gap in determining optimal intervention strategies for holistic child health promotion.

In this study, we used three different exercise interventions (PA, CT, and PA coupled with CT) to evaluate their effects on physical fitness, cognition, and mental health in children aged 9 to 10 years old. This randomized controlled trial used three distinct intervention protocols to systematically compare their effects on school-aged children: (1) PA: structured aerobic/resistance training, (2) CT: computerized attention/memory tasks, and (3) combined PA+CT: integrated physical-cognitive dual-task program. Therefore, the first purpose was to quantify the differential impacts of these interventions on physical fitness, cognitive function, and mental health indicators in 9–10-year-olds and qualitatively analyze participants’ perceived benefits, engagement patterns, and motivational drivers across intervention modalities. The secondary purpose was to compare the effect size of PA+CT versus single-domain interventions (PA-only/CT-only) through standardized mean difference (SMD) calculations. The working hypothesis was that, as compared to either PA or CT, the group simultaneously receiving PA and CT programs would demonstrate significantly greater improvement in physical performance and cognitive measures.

## 2 Materials and methods

### 2.1 Experiment design and participants

This study utilized G*Power software to calculate the required sample size. To ensure adequate statistical power for detecting intergroup differences, a minimum of 120 participants was determined by considering medium to large effect sizes (Cohen’s d = 0.5–0.8) based on previous research (Lubans et al., 2016; Hillman et al., 2014) involving repeated sprint protocols integrating physical activity (PA) and cognitive training (CT). To mitigate potential data loss due to participant dropout, the study ultimately recruited 150 individuals. Using convenience sampling, a total of 150 healthy children (80 boys and 70 girls) aged between 9 and 10 years from a primary school in Beijing were recruited to participate in this study.

**Figure 1:**
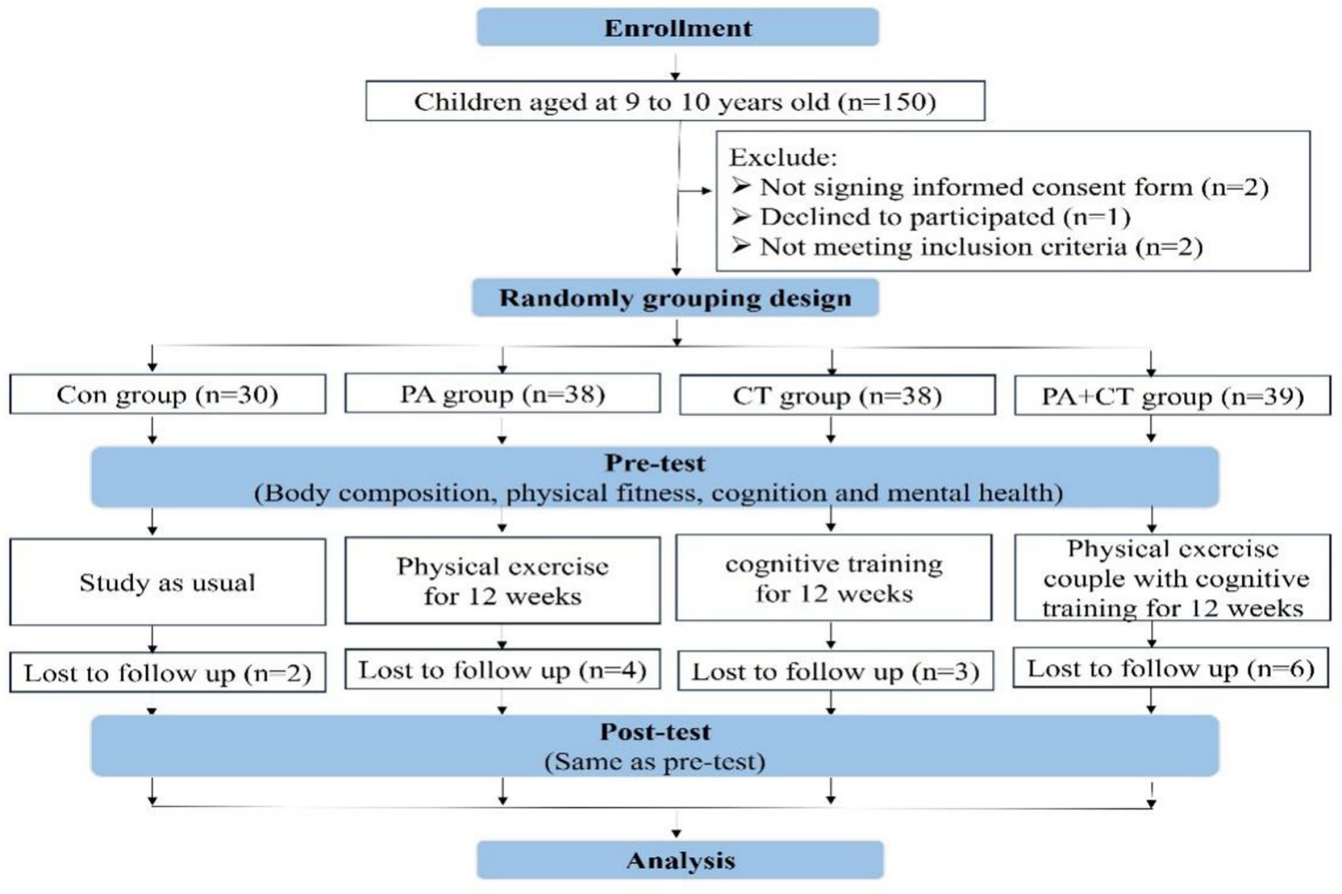
Schematic of the experimental protocol.

This study included 150 healthy children (80 boys and 70 girls) aged between 9 and 10 years old from a primary school in Beijing using a convenience sample. It is a randomized controlled trial designed to compare the effects of PA and CT on cognitive performance, physical fitness, and mental health in children. Finally, 145 right-handed children from China (boys = 78 and girls = 67) participated in our study and were cluster-randomly allocated into four groups: Con group (n = 30), PA group (n = 38), CT group (n = 38), and PA+CT group (n = 39). Except for the Con group, all of the children received 12 weeks of PA or (and) CT interventions. The full set of outcomes (e.g., body composition, physical fitness, and cognitive function tests) was assessed twice: immediately before and after the 12-week exercise interventions. At the end of the experiment, 130 children had completed the whole experiment, and their results were considered effective samples (Con group, n = 28; PA group, n = 34; CT group, n = 35; and PA+CT group, n = 33). More details in this study can be found in Figure 1. To avoid any potential bias in the assessment process, the assessors did not participate in the intervention delivery or group allocation. In addition, we ensured protocol adherence by implementing standardized procedures, monitoring provider training, and conducting regular audits to maintain intervention fidelity. Inclusion criteria: (a) ages 9–10 years; (b) right-handed; (c) no physical disabilities, neurological disorders, or cognitive impairment; (d) no surgical interventions or pharmacological treatments within 6 months prior; and (e) no PA organized by other institutions in their spare time. All participants and legal guardians provided written informed consent after receiving full disclosure of study procedures. The experiment in this study was approved by the Human Research Ethics Committee of Capital University of Physical Education and Sports (2020A73).

### 2.2 Intervention program for different exercise ability development

All intervention groups (PA, CT, and PA+CT) completed at least four (of five) supervised exercise sessions per week. The duration of each exercise lasted 40 min daily, four times per week for 12 weeks, and the duration and intensity of the interventions were shown to be sufficient to elicit measurable changes in physical and mental health outcomes (Hao et al., 2023). In the PA group, the children mainly received PA interventions (40 min/time). The physical fitness intervention for children was created using the ideas of enjoyment, novelty, and challenge, and it was adapted to the features of preschoolers by including gamification aspects, such as balance beam, crab crawling, and multi-directional movement. Details as Table 1 (Aly et al., 2024). In the CT group, the children were allowed to randomly choose two cognitive games (20 min each) at random from four different games (40 min), including instantly remember the stars game, typing game, transient memory game, and discerning the direction of arrow movement game, as detailed in Table 2. In the PA+CT group, the children received twice as much PA from Table 1 and CT twice per week from Table 2. The Supplementary materials provide a detailed description of PA and CT protocols. Six specialized physical education instructors implemented the curriculum, and attendance records were maintained for all sessions.

**Table 1:** PA schedule.

Training time	Training content	Example	Training frequency
1–4 weeks	Balance, aerobic capacity	Drop the handkerchief	10–20 min/game; two games/session; four sessions/week
		Relay run	
		Grass skiing	
		Balance beam	
4–8 weeks	Speed and core stability	Obstacle jump	10–20 min/game; two games/session; four sessions/week
		Crab crawling	
		throw the bean bag	
8–12 weeks	Reactivity and flexibility	Go/no go	10–20 min/game; two games/session; four sessions/week
		Multi-directional movement	
		Turn left, turn right	
		Do the action as instructed by the command	

**Table 2:** CT schedule.

Training time	Training content	Example	Training frequency
1–4 weeks	Attention, thinking ability	Discerning the direction of arrow movement	10–20 min/game; two games/session; four sessions/week
		Hanoi tower	
		Attention concentrativeness	
		Attentional blink	
4–8 weeks	Reactive time, perception	Perception time	10–20 min/game; two games/session; four sessions/week
		Red and blue	
		Left–right	
		Instantaneous memory	
8–12 weeks	Memory, cognitive flexibility	Transient memory game	10–20 min/game; two games/session; four sessions/week
		Repeated numbers and letters	
		Memory allocation	
		Card-sorting	

### 2.3 Morphological tests

The morphological tests performed in this study measured both height and weight. Height and weight were measured independently on scales with a 0.1 cm precision. BMI values were converted to percentiles (weight in kilograms divided by height in meters squared).

### 2.4 Physical fitness tests

Physical fitness indicators in this study include vital capacity, flexibility quality, speed quality, aerobic performance, and physical coordination, according to the National Physical Fitness Standards Manual - Children Part (State Sport General Administration, 2003).

#### 2.4.1 Vital capacity (Milliliter; mL)

During the test, the participant held the handle and inhaled as hard as they could then placed their mouth over the air inlet of the pneumatometer and slowly exhaled until the lung capacity value displayed on the pneumatometer screen did not increase. Repeat twice and record the maximum value.

#### 2.4.2 Sit and reach (Centimeters; cm)

Sit and reach was used to assess flexibility quality. After removing their shoes, participants sit and bend forward, such that the full soles of both feet make contact with the sit-and-reach meter, the knees are straightened, and both hands are as close to the measurement device as possible. Two measurements are done, with the best result recorded at 0.1 cm.

#### 2.4.3 50 m run (Seconds; s)

A 50-m run test was performed to assess speed quality. The participant stood in front of the starting line, and when the command “Ready-Go” was heard, the participant was asked to run as fast as possible to the end. Note that the start and finish are 50 meters apart.

#### 2.4.4 Rope skipping (Times, t)

Rope skipping was used to evaluate aerobic performance. The participant stands naturally, with slightly staggered ankles, and stares in front. The upper arm is near the body, and the wrist shakes the rope. The participant was required to rope skip as soon as possible. Then record the number of skipping ropes in 1 min.

#### 2.4.5 Sit-up (Times, t)

Sit-ups were used to evaluate physical coordination. Participants lie on a mat, knees bent at 90°. They sit up by raising their upper body to contact the knees with the elbows on both sides, then returning to their original position. This maneuver is completed in 60 s and is recorded repeatedly.

### 2.5 Cognitive function tests

Cognitive function tests were adopted before and after 12 weeks of exercise training. Cognitive function tests include attention, simple reaction time, and spatial memory (Hao et al., 2023).

#### 2.5.1 Attention

The Schulte Table test was used to evaluate attention and exchange stability. The Schulte Table test uses 3 × 3 squares with numbers from 1 to 9 in a grid. The children were asked to rank from 1 to 9 as quickly as possible, and the time spent was recorded. Shorter completion times during this assessment correlate with enhanced attentional performance. If the click sequence is incorrect, a tone will appear until the participant finds the correct sequence and clicks. After three consecutive tests, the shortest value was the attention span time.

#### 2.5.2 Simple reaction time

The red-green test is used to reflect the rapid response ability of children after receiving a fixed and single visual stimulus. During the test, the visual stimulus was a red circle, and when the red circle stimulus changed to green in the middle of the screen, the participant had to press the green button to respond as soon as possible, and the reaction time was recorded. A total of 30 tests were performed, with each interval of 2 s. If there is a preemptive keystroke, the test result is invalid, and the computer sounds a warning tone.

#### 2.5.3 Spatial memory

**Figure 2:**
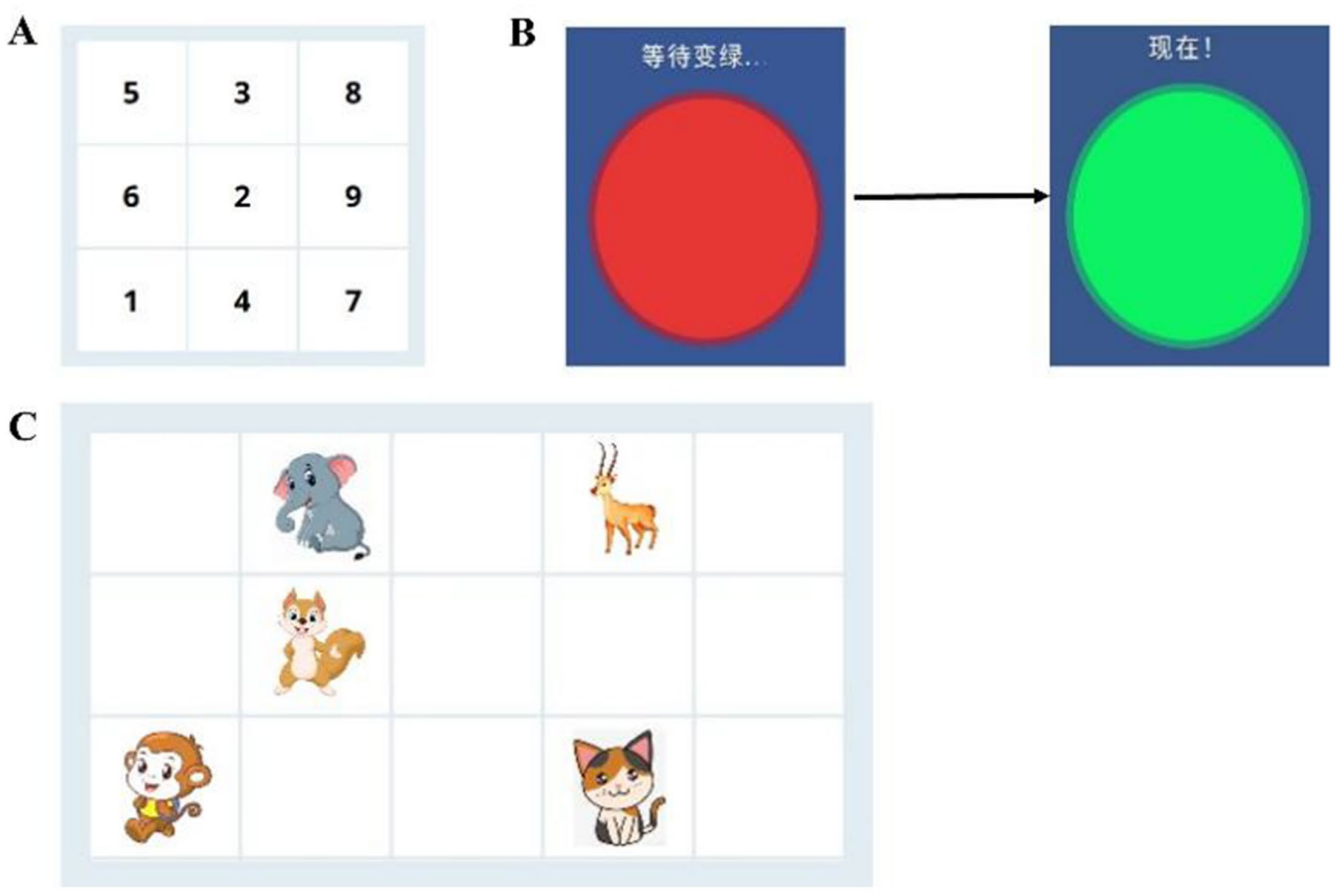
Diagrammatic representation of cognition tests. **(A)** The Schulte Table test. **(B)** Red-green test. **(C)** The spatial location memory span test.

The spatial location memory span test was used to test spatial orientation perception and short-term memory. In the test, a 5*3 grid was shown on the computer screen. Some animals would emerge from these 15 divisions randomly (which started with a continuous present three divisions). The participant was required to remember the location and order in which the animal appeared and then click on the squares in the order in which the animals had just appeared as much as possible and then click the “NEXT” button to enter. The test would not terminate until either three consecutive incorrect responses were received or 12 assigned tasks were completed (Figure 2).

### 2.6 Mental health tests

The mental health level of the students was measured before and after exercise interventions, which were measured by the following parameters. The reliability and validity of the scales were tested, and the results showed internal consistency and validity in normal populations. The Cronbach’s alpha reliability coefficient for the SHS in our study was 0.85. Our and other studies support that such scales are reliable measures that could be used in children’s emotion research (Rodriguez-Ayllon et al., 2018; Extremera and Fernández-Berrocal, 2014).

The State–Trait Anxiety Inventory (STAI-C) for Children assessed childhood trait anxiety (Aly et al., 2019). The STAI-C is one of the most often used measures of general anxiety. The STAI-C assesses concern, tension, apprehension, and uneasiness. 20 It is a 20-item self-administered instrument with categories ranging from 1 (nearly never) to 3 (frequently). The scores range from 20 to 60. Higher scores indicate higher trait anxiety levels.

The Children’s Depression Inventory (CDI) was used to determine the severity of depression (Hao et al., 2023). The CDI’s 27 items were classified into five factor areas: negative emotions, interpersonal issues, inefficiency, anhedonia, and negative self-esteem, with a global score used for analysis in the study. The answer ranges from 0 to 2. The final score is calculated by adding the scores of 27 elements, which range from 0 (lowest depression level) to 54 (highest depression level).

Positive and negative emotions in children were measured using the Positive and Negative Affect Schedule for Children (PANAS-C) (Aly et al., 2019). PANAS-C contains 20 questions with answers ranging from 1 to 3. Negative emotions were calculated as the sum of 10 components. Final scores range from 10 to 30. The higher the score, the greater the negative.

Happiness was assessed using the Subjective Happiness Scale (SHS) (State Sport General Administration, 2003). It consists of four questions, with responses ranging from 1 to 7. The final score is the total of the first three components, with values ranging from 3 (least happiness) to 21 (most happiness).

### 2.7 Statistical analyses

Statistical procedures were performed using the SPSS 22.0 software and GraphPad Prism 8.0 software. The sample of this study is presented as means and standard deviations (SDs). G*Power statistical analysis was used to evaluate the sample size in this experiment. The paired sample *t*-test was used for within-group comparisons, while the one-way analysis of variance was used for multiple group comparisons at the same period. A significant difference level of *p <* 0.05 was set.

## 3 Results

### 3.1 Descriptive baseline characteristics in each group

Baseline sample characteristics for all of the children in each group are shown in Table 3. There were no significant differences among the groups at the pre-test stage, such as height, weight, and BMI (*p* > 0.05).

**Table 3:** Descriptive baseline characteristics of the participants meeting the per-protocol criteria.

Baseline characteristics	Con	PA	CT	PA+CT
Age [year]	9.86 ± 0.54	9.63 ± 0.36	9.49 ± 0.26	9.59 ± 0.42
Height [cm]	139.14 ± 2.7	139.57 ± 2.3	140.63 ± 3.84	139.28 ± 1.86
Weight [kg]	32.17 ± 4.17	31.50 ± 3.17	32.71 ± 4.28	31.23 ± 2.35
BMI [kg/m^2^]	16.65 ± 2.39	16.18 ± 1.68	16.55 ± 2.1	16.11 ± 1.25

There were no significant differences among each group. Data are expressed as mean ± standard deviation or %. The paired sample *t*-test was used for within-group comparisons, and the one-way analysis of variance was used for multiple group comparisons at the same period. “Con” represents the “Con” group; “PA” represents the physical activity group; “CT” represents the cognitive training group; and “PA+CT” represents the physical activity coupled with the cognitive training group. “BMI” represents the body mass index.

### 3.2 Effect of three exercise types interventions on physical fitness in each group

**Figure 3:**
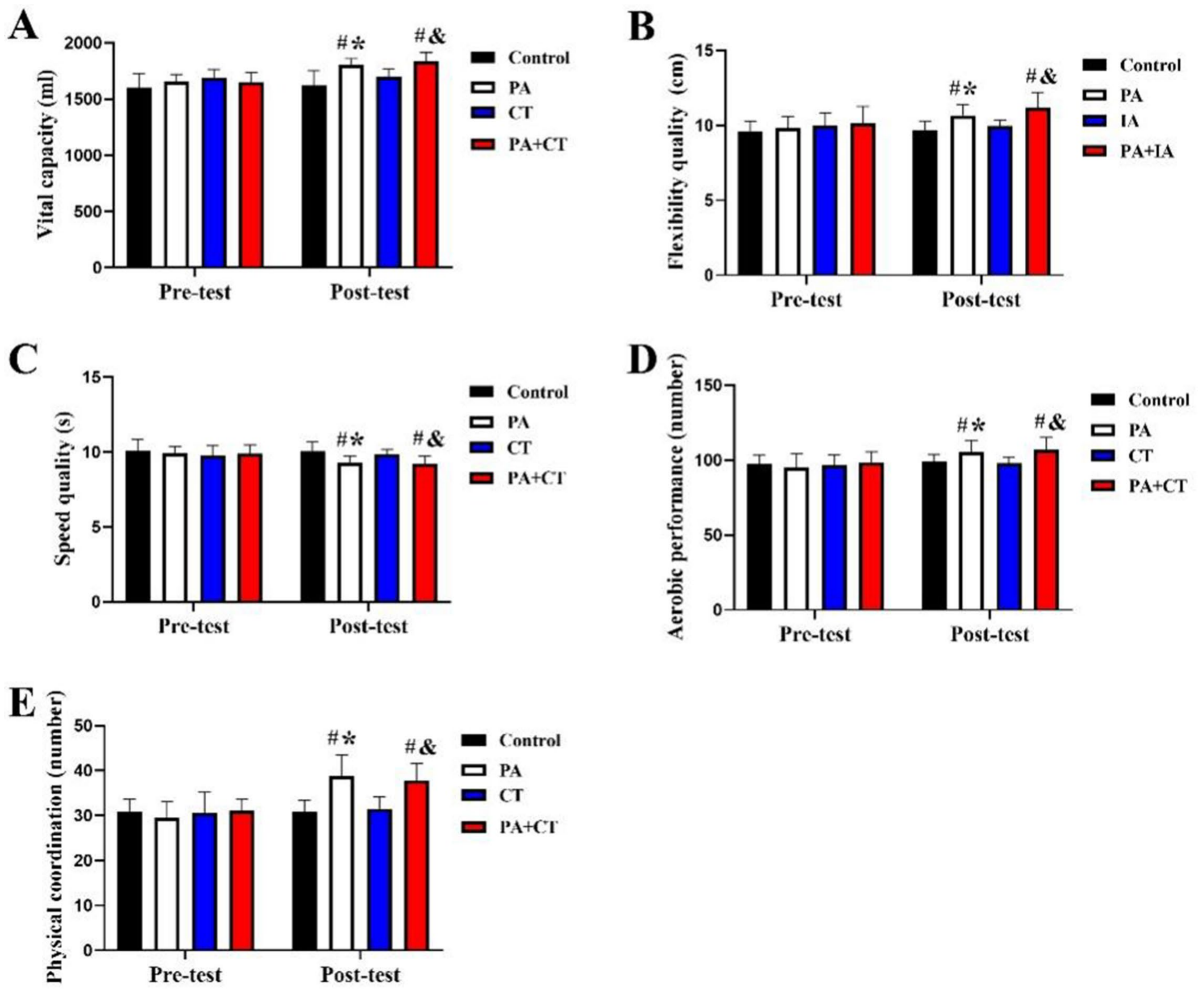
Effect of three exercise types interventions on physical fitness parameters in children. **(A)** Vital capacity, **(B)** flexibility quality, **(C)** speed quality, **(D)** aerobic performance, and **(E)** physical coordination. ^#^*p* < 0.05 represents comparisons with Con in the post-test stage. ^*^p < 0.05 represents pre-test vs. post-test in the PA group; ^&^*p* < 0.05 represents pre-test vs. post-test in the PA+CT group.

The validation analysis of three exercise types interventions on physical fitness in school-aged children is presented in Figure 3. We can learn by comparison among groups that there were no differences in terms of physical fitness parameters among these groups (*p* > 0.05) at the pre-test stage, including vital capacity, flexibility quality, speed quality, aerobic performance, and physical coordination. At the post-test stage, there were significant differences among the four groups in terms of physical fitness parameters (*p* < 0.05). When compared with control group, significantly improvement of physical fitness parameters (vital capacity, flexibility quality, speed quality, aerobic performance, and physical coordination) in the PA and PA+CT groups (*p* < 0.05; PA [vital capacity: +11.14%; *F* = 3.097 (28, 34), *t* = 3.36; flexibility quality: +10.45%; *F* = 3.61 (28, 34), *t* = 2.992; speed quality: −7.53%; *F* = 2.576 (28, 34), *t* = 2.992; aerobic performance: +6.51%; *F* = 4.02 (28, 34), *t* = 2.992; physical coordination: +25.91%; *F* = 2.281 (28, 34), *t* = 4.09], PA+CT [vital capacity: +13.12%; *F* = 4.92 (28, 33), *t* = 4.19; flexibility quality: +15.89%; *F* = 3.93 (28, 33), *t* = 4.03; speed quality: −8.19%, *F* = 4.01 (28, 33), *t* = 6.19; aerobic performance: +7.93%; *F* = 4.9 (28, 33), *t* = 2.57; physical coordination: +22.70%; *F* = 5.1 (28, 33), *t* = 4.21]), but no significantly difference in the CT group (*p* > 0.05).

Intragroup comparison in Figure 3 demonstrated that there was no significant difference in physical fitness parameters from pre- to post-intervention in the Con group (*p* > 0.05). In addition, there was a significant improvement of physical fitness parameters in pre- to post-intervention in both the PA and PA+CT groups (*p* < 0.05; PA [vital capacity: +8.89%; *F* = 4.62 (34, 34), *t* = 2.36; flexibility quality: +8.70%; *F* = 3.80 (34, 34), *t* = 2.10; speed quality: −6.23%; *F* = 4.27 (34, 34), *t* = 2.992; aerobic performance: +10.56%; *F* = 6.21 (34, 34), *t* = 2.08; physical coordination: +31.81%; *F* = 4.09 (34, 34), *t* = 4.09]; PA+CT [vital capacity: +11.12%; *F* = 5.67 (33, 33), *t* = 3.08; flexibility quality: +10.32%; *F* = 4.62 (33, 33), *t* = 2.99; speed quality: −6.62%; *F* = 4.51 (33, 33), *t* = 3.61; aerobic performance: +8.44%; *F* = 6.30 (33, 33), *t* = 4.16; physical coordination: +21.99%; *F* = 4.36 (33, 33), *t* = 5.98]), but no significantly improvement effect in the CT group in the pre-test and post-test (*p* > 0.05). It revealed that PA, PA coupled with CT can significantly improve physical fitness parameters in children, such as. However, CT alone showed no significant impact on the physical fitness aspect.

### 3.3 Effect of three exercise types interventions on cognition in each group

**Figure 4:**
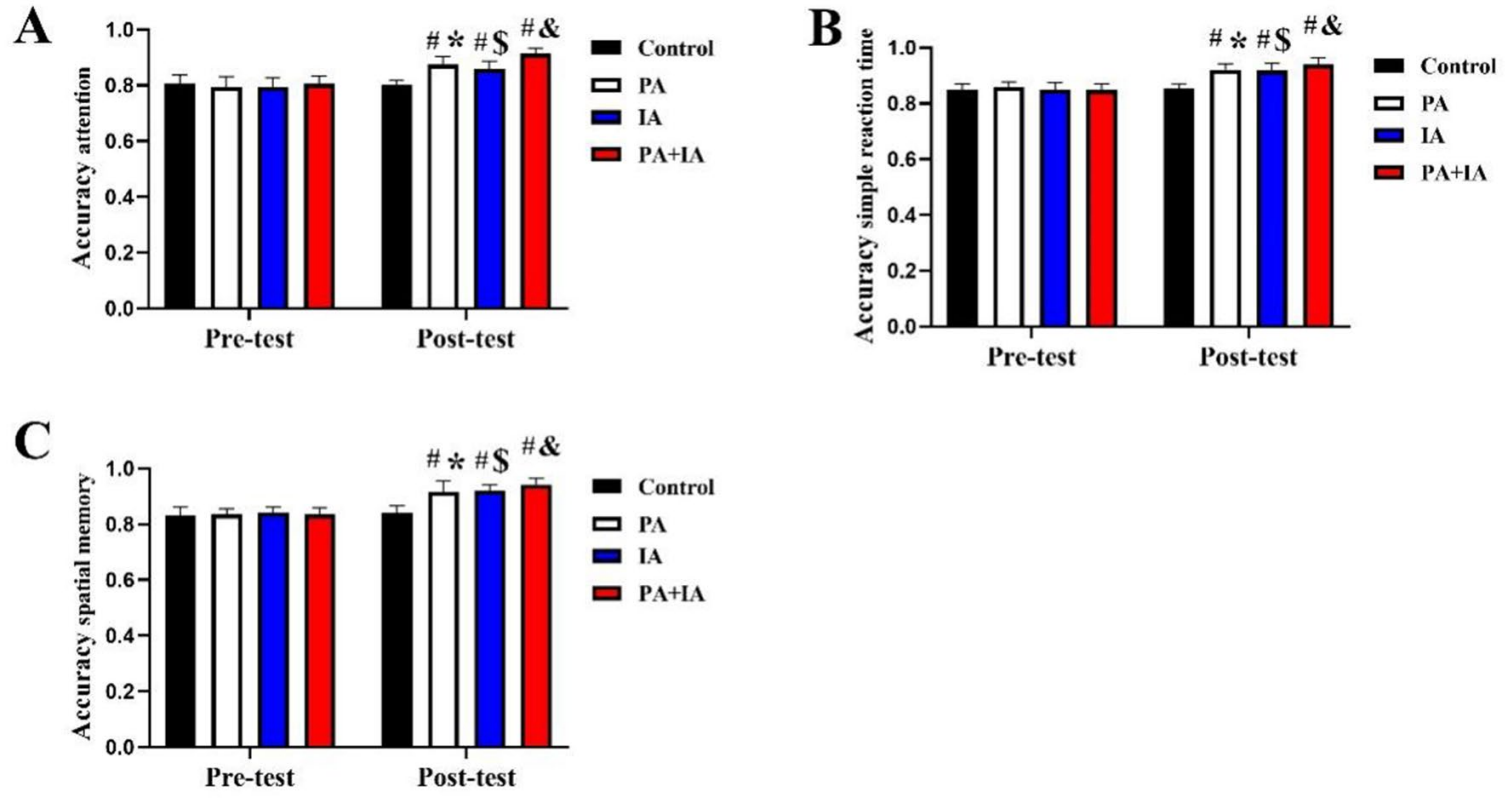
Effect of three exercise types interventions on cognition parameters in children. **(A)** Attention, **(B)** simple reaction time, and **(C)** spatial memory. ^#^*p* < 0.05 represents comparisons with Con in the post-test stage. ^*^p < 0.05 represents pre-test vs. post-test in the PA group; ^$^*p* < 0.05 represents pre-test vs. post-test in the CT group, ^&^*p* < 0.05 represents pre-test vs. post-test in the PA+CT group.

Figure 4 shows the distribution of cognition among the four groups. In the pre-test stage, there was no significant difference in cognition parameters among each group (*p* > 0.05), such as attention, simple reaction time, and spatial memory (*p* < 0.05). However, significant improvement of all cognition parameters in the PA, CT, PA+CT groups than that in the control group in the post-test stage (*p* < 0.05; PA [attention: +7.10%; *F* = 3.21 (28, 34), *t* = 4.61; simple reaction time: +7.95%; *F* = 4.39 (28, 34), *t* = 3.92; spatial memory: +9.57%; *F* = 3.72 (28, 34), *t* = 4.71], CT [attention: +5.97%; *F* = 3.57 (28, 35), *t* = 5.504; simple reaction time: +6.93%; *F* = 4.50 (28, 35), *t* = 5.504; spatial memory: +10.84%; *F* = 4.21 (28, 35), *t* = 5.02], PA+CT [attention: +11.95%; *F* = 3.99 (28, 33), *t* = 8.52; simple reaction time: +10.75%; *F* = 5.02 (28, 33), *t* = 6.21; spatial memory: +12.82%; *F* = 4.60 (28, 33), *t* = 9.07]), and the improvement effect of cognition in the PA+CT group is better than the PA and CT groups (*p* < 0.05).

We also demonstrated that there was no significant difference in the mental health index from pre- to post-intervention in the Con group (*p* > 0.05). In addition, there was a significant improvement of cognition parameters (attention, simple reaction time, spatial memory) in pre- to post-intervention after 12 weeks PA regarding intra-group comparison (*p* < 0.05; PA [attention: +8.96%; *F* = 2.57 (34, 34), *t* = 2.31; simple reaction time: +6.34%; *F* = 2.67 (34, 34), *t* = 3.11; spatial memory: +10.06%; *F* = 3.32 (34, 34), *t* = 4.29], CT [attention: +7.63%; *F* = 3.59 (35, 35), *t* = 3.41; simple reaction time: +10.44%; *F* = 3.43 (35, 35), *t* = 3.88; spatial memory: +10.71%; *F* = 4.01 (35, 35), *t* = 5.59], PA+CT [attention: +12.77%; *F* = 4.56 (33, 33), *t* = 5.504; simple reaction time: +7.10%; *F* = 4.99 (33, 33), *t* = 8.52; *t* = 6.50; spatial memory: +12.26%; *F* = 5.61 (33, 33), *t* = 10.75]. Similarly, post-test cognition parameters of the PA, CT, and PA+CT groups were then compared to the pre-test (*p* < 0.05). The results of this study showed that three exercise types interventions, including PA, CT, and PA combined with CT, can all improve cognition in children.

### 3.4 Effect of three exercise types interventions on mental health in each group

**Figure 5:**
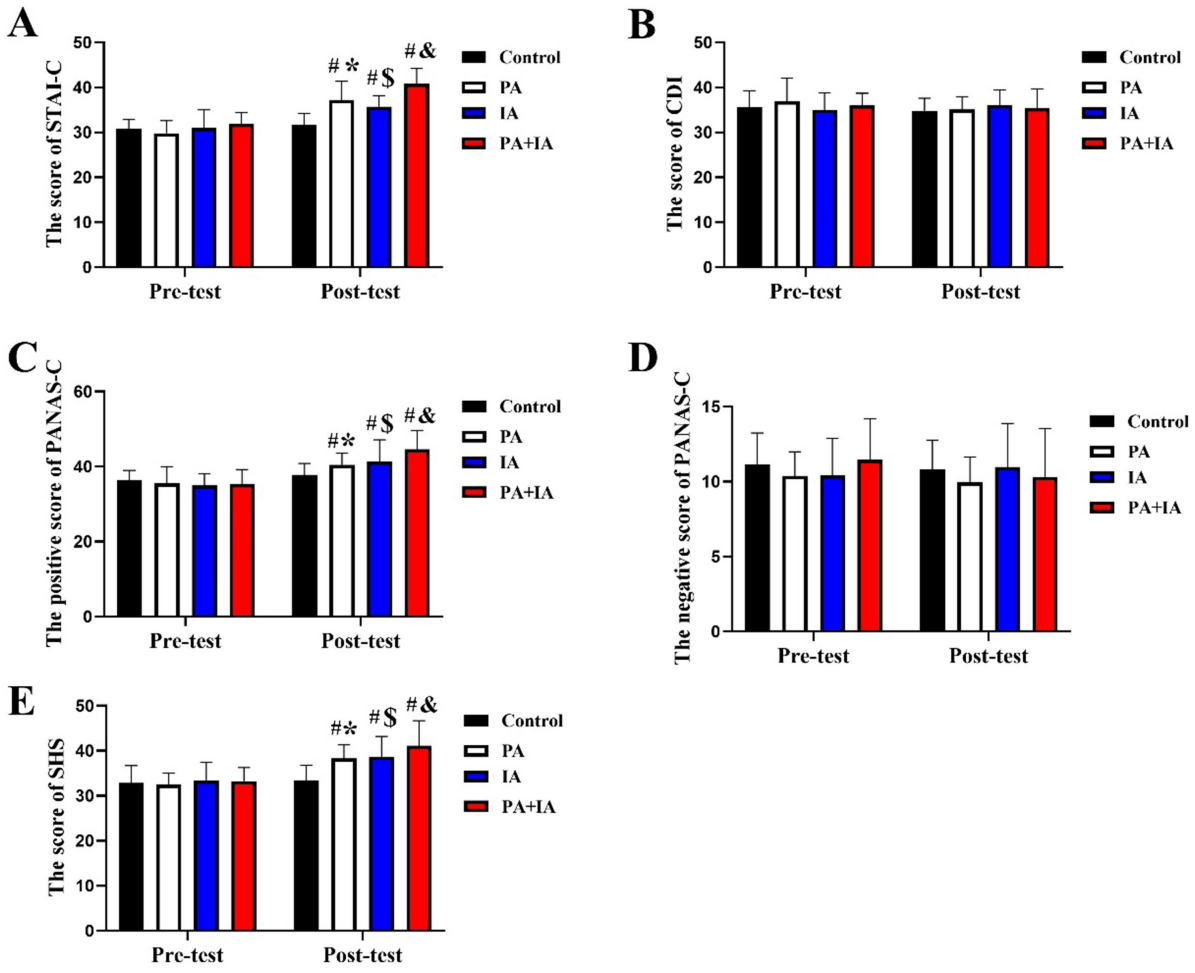
Effect of three exercise types interventions on mental health in children. **(A)** The score of STAI-C, **(B)** the score of CDI, **(C,D)** the positive and negative score of PANAS-C, and **(E)** the score of SHS. ^#^*p* < 0.05 represents comparisons with Con in the post-test stage. ^*^p < 0.05 represents pre-test vs. post-test in the PA group; ^$^*p* < 0.05 represents pre-test vs. post-test in the CT group, ^&^*p* < 0.05 represents pre-test vs. post-test in the PA+CT group.

Figure 5 shows the variation in mental health after three exercise types interventions among each group. Figure 4 shows that there were no significant differences among the four groups in mental health parameters (anxiety, depression, positive/negative emotions, and happiness) during the pre-test stage (*p* > 0.05). In the post-test stage, mental health outcomes, including reduced anxiety and increased positive emotions, were significantly better in all intervention groups ((*p* < 0.05; PA [STAI-C: +17.05%; *F* = 5.52 (28, 34), *t* = 2.25; the positive of PANAS-C: +7.11%; *F* = 5.67 (28, 34), *t* = 2.6; SHS: +14.52%; *F* = 6.25 (28, 34), *t* = 4.81], CT [STAI-C: +12.45%; *F* = 3.57 (28, 35), *t* = 1.89; the positive of PANAS-C: +9.57%; *F* = 3.80 (28, 35), *t* = 3.9; SHS: +15.60%; *F* = 4.23 (28, 35), *t* = 3.76], PA+CT [STAI-C: +28.32%; *F* = 6.37 (28, 33), *t* = 3.56; the positive of PANAS-C: +18.13%; *F* = 5.98 (28, 33), *t* = 4.7; SHS: +22.97%; *F* = 6.89 (28, 33), *t* = 6.21])), with the strongest effects in PA+CT. Additionally, children in the PA+CT group also performed better on mental health parameters than those in the PA and CT groups (*p* < 0.05), but no significant improvement effect in the remaining negative score of PANAS-C in the post-test (*p* > 0.05).

Intragroup comparison also demonstrated that there was no significant difference in the mental health index between pre-test and post-test stages in the Con group (*p* > 0.05). The PA group showed a substantial increase in the mental health index at the post-test stage in the PA group compared with the pre-test stage [*p* < 0.05; STAI-C: +24.75%; *F* = 4.27 (34, 34), *t* = 1.40; the positive of PANAS-C: +13.95%; *F* = 4.61 (34, 34), *t* = 2.31; SHS: +18.03%; *F* = 5.61 (34, 34), *t* = 3.32]; this phenomenon also can be found in the CT and PA+CT groups (*p* < 0.05; CT [STAI-C: +15.30%; *F* = 4.78 (35, 35), *t* = 1.89; the positive of PANAS-C: +18.38%; *F* = 3.91 (35, 35), *t* = 3.98; SHS: +16.02%; *F* = 4.96 (35, 35), *t* = 3.76], PA+CT [STAI-C: +27.95%; *F* = 3.92 (33, 33), *t* = 3.56; the positive of PANAS-C: +26.33%; *F* = 4.17 (33, 33), *t* = 4.7; SHS: +23.68%; *F* = 4.51 (33, 33), *t* = 6.21]. This result revealed that three exercise types interventions can all improve mental health in children, including PA, CT, and PA combined with CT.

## 4 Discussion

This randomized controlled trial systematically evaluated the comparative efficacy of three intervention modalities—PA, CT, and combined (PA+CT)—on multidimensional health outcomes (physical fitness, cognitive performance, and mental health) in children aged 9–10 years. The key findings are as follows: (1) All three interventions demonstrated measurable benefits across the target domains, with effect sizes ranging from small to moderate. (2) The PA+CT group exhibited superior outcomes compared to single interventions, particularly in physical fitness, cognition, and emotional regulation. Participant feedback indicated high engagement and perceived enjoyment for the combined protocol. It also directly demonstrated that it is a good way to combine PA and CT programs in the future children’s curriculum.

In this study, we first collected baseline sample characteristics in each group to avoid the influence of body composition on experimental results. This data revealed that there were no significant differences in anthropometric measurements among each group in the pre-test stage, such as height, weight, and BMI. It revealed that the body composition was similar in each group. In terms of physical fitness, the pre-post values exhibited significant improvements in the physical fitness test in the PA and PA+CT groups, the improvements in physical fitness parameters, including vital capacity, flexibility quality, speed quality, aerobic performance, and physical coordination. The results directly revealed that PA, PA coupled with CT, can significantly improve physical fitness parameters in children, such as. There are various evidence that directly proves the positive effect of PA on physical fitness in school children under normal or disease status (Wu et al., 2023, Wang et al., 2023, Yu et al., 2018). Our study also demonstrated that PA coupled with CT significantly improves physical fitness in children, which is consistent with previous studies (Robinson et al., 2022). It is probably because PA coupled with CT intervention is a diversified and attractive program. This article further revealed that PA with or without cognitive intervention can promote both physical fitness and cognitive development in children. Consistent with this result, our study also demonstrated that CT alone demonstrated no significant impact on athletes’ physical fitness. There is probably that CT mainly focused on mental activities, but had no significant effect on physical fitness. Thus, three exercise types interventions significantly improved physical fitness in children, including PA, PA coupled with CT. These results proved the importance of comprehensive PA and CT on physical fitness in children, but the dose-dependent relationships are needed to be further studied.

We next explored the effect of three exercise types interventions on cognition in children. The results of the cognition test in our study showed that when compared with the con group, the cognitive index (attention, simple reaction time, spatial memory) was significantly better in the PA and CT groups, demonstrating that grade 3–4 students who received PA or CT showed better cognition level than control students. A cross-sectional study found a positive effect of PA on cognition in children, as previously reported. This article also explicitly stated that cognition acts as a mediator in the relationship between PA and academic achievement (Visier-Alfonso et al., 2021). Interestingly, it has been shown that higher aerobic fitness was associated with shorter response time and higher response accuracy as well as a more efficient executive network in the attention network test (Abdelkarim et al., 2023). Similarly, a cross-sectional study was reported by Aly et al. (2024) that greater aerobic fitness was related to higher academic outcomes and greater maximum grip strength was related to a shorter response time in a cognitive-related task (Aly et al., 2024). Aly et al.’s study provides neurophysiological evidence that regular PA is positively associated with better neural efficiency in Stroop (i.e., color-naming) and reverse Stroop (i.e., word-meaning) tasks (Aly and Kojima, 2020). The cognition results in our study demonstrate for the first time that the improvement effect of PA coupled with CT is better than the single PA or CT intervention. As described above, PA coupled with CT intervention is a diversified and attractive program. The event-related potential results provided a deeper understanding that exercise interventions, regardless of the exercise type, are associated with a larger amount of neural attentional resources and faster stimulus evaluation speed (Aly et al., 2019). Together, our study and previous research collectively bridge the knowledge gap on the associations of exercise interventions to physical fitness and cognition in children. Interestingly, our study demonstrated that PA coupled with CT is a better way to improve cognitive outcomes than single PA or CT.

Finally, we also discussed the effect of three exercise types interventions on mental health in children. The present study observed a significant improvement in the mental health index in the PA, CT, and PA+CT groups when compared with the control group, indicating that the children performed better on the mental health level after receiving different types of exercise, including PA, CT, and PA coupled with CT. Jiang R’s article also confirmed the positive effect of PA on mental health among children participating in sport-specific training (Jiang et al., 2021). A systematic review performed by Hale et al. (2023) also demonstrated that PA interventions enhance the psychological wellbeing of children in school (Hale et al., 2023). However, Migueles’s experiment showed that 20 20-week exercise program did not improve mental health in children with overweight or obesity (Migueles et al., 2023). Differences between our findings and earlier research could be explained by the exercise program’s heterogeneity (type: single exercise vs. varied exercise), the study sample’s characteristics (sex, weight status), the study design, and so on. The results in our study also pointed out that there was no significant improvement in the remaining negative score of PANAS-C in the post-test. This seems partly owing to children still being young, with high levels of well-being and low levels of ill-being, thus further improving these outcomes is needless and complex. Thus, three exercise types interventions can all improve mental health in children, including PA, CT, and PA combined with CT, and the improvement effect of PA coupled with CT on mental health in children is better than single PA or CT.

Together, compared with traditional exercise programs with a single project and action form, comprehensive exercise programs with multiple action forms, including but not limited to PA coupled with CT, can better improve the physical fitness and mental health in children. Our findings may provide some guiding suggestions in terms of the arrangement of sports activity content. Given these findings, we recommend that in the future, authorities and physical educators promote PA coupled with CT intervention for children and adolescents in physical education classes and amateur sports activities. Our findings might be limited by the long-term effects of three exercise types interventions on physical and mental health in children, which will need to be confirmed in the future, with an adequately sized sample and powerful randomized controlled trials. In addition, we should consider the influence of parents’ education and economic level on the results of the experiment. A clinical trial is also planned to extend the present application to include PA coupled with CT.

## 5 Conclusion

Three exercise types programs designed for primary school physical education have positive effects on the physical and mental health of children aged 9 to 10 years old. Compared with the exercise programs with a single project and action form, comprehensive exercise programs with multiple action forms, including but not limited to physical activity coupled with cognitive training, are more effective in improving physical and mental health in children. These findings provide empirical support for developing multidimensional physical education curricula to optimize child development.

## Data Availability

The raw data supporting the conclusions of this article will be made available by the authors, without undue reservation.
