# Supplementary material for: Comparing the effects of physical activity and cognitive training on cognitive performance, physical fitness, and mental health in 9- to 10-year-old children: a randomized clinical trial
Source: Front Psychol. 2025 Jun 26;16:1555451. doi: 10.3389/fpsyg.2025.1555451 (PMC12241119; doi:10.3389/fpsyg.2025.1555451)
Supplement: Supplementary file 2 [file Table_2.docx]

**表1 身体活动计划表**

| **训练时间** | **训练内容** | **举例/时间** | **详细步骤** |
| --- | --- | --- | --- |
| 1-4 week | 平衡，有氧能力 | 丢手绢  (15-20min) | 所有儿童围坐成一个圆圈，第一个丢手绢的人可以是自荐或由大家推选。 3. 当大家齐声唱起《丢手绢》儿歌时，丢手绢的人会随机将手绢丢在某个玩家的身后。 被丢手绢的玩家需立刻起身追赶丢手绢的人。 如果丢手绢的人能在儿歌结束前回到原位置坐下，则被丢手绢的玩家淘汰。 |
|  |  | 准备-跑  (10-15min) | 当教师发出“准备”指令时，儿童做好准备跑步的姿势；当儿童听到“跑”的指令时，立即出发跑50米。 |
|  |  | 滑草  (15-20min) | 在室外找一处草坪斜坡，铺上塑料布，让幼儿由上滑下来。或是让幼儿坐在纸箱内或盆内，由上滑下来。也可用大塑料布铺在草坪斜坡上，当幼儿由上滑下时，大人可以水柱冲他的身体，增加触觉及趣味性。 |
|  |  | 平衡木  (10-15min) | 将平衡木横向放置,让幼儿先用手辅助跨越平衡木。幼儿熟悉动作后,以不用手辅助的方式跨过。慢慢增加跨越的速度,并增加单脚跳越的项目。此外,可以让幼儿将两手放在平衡木上,挑战用手撑起跳跃的动作。 |
| 4-8 week | 速度，核心 | 障碍跑  (15-20min) | 儿童择一根塑料棍和两个相同的皮鞋盒，尝试跳过有一定高度的障碍物。接下来，儿童可选择原来高度的障碍物进行垮跳，也可选择更高障碍物垮跳。 |
|  |  | 螃蟹爬行  (10-15min) | 播放《螃蟹体操》音乐，所有儿童一起跟着音乐跳螃蟹操 |
|  |  | 扔沙包  (10-15min) | 让孩子先把较小的纸团往远处丢，然后换上稍大的纸团再丟，然后再换成沙包丢，反复练习。 |
| 8-12 week | 反应，灵活性 | 走/不走  (15-20min) | 被试需要在听到的语音提示来决定是跑步还是不跑步，当受试者听到“跑“的指令不跑，相反，当听到”不跑“的指令时则跑步看到图片时需要依据单词结尾的字母是“R”还是“N”来决定是否要进行按键，即单词结尾的字母是“R”就按键，单词结尾的字母是“N”就不按键。 |
|  |  | 多方向移动  (15-20min) | 直线方向移动：包括向前、向后、向左、向右等四个方向的快速移动。  曲线方向移动：包括弧形、圆形、椭圆形等三个方向的快速移动。 |
|  |  | 向左-右走  (10-15min) | 孩子举起双手与耳朵齐高，双脚略弯曲、，往左和往右连续横行，如螃蟹走路状。或者双手轻轻放下，侧着头，踏脚向前、向左、向右走。也可双手平举向前，或伸开摆放在两侧，踏脚向前、向左、向右走。 |
|  |  | 按口令做动作  (10-15min) | 游戏开始前，规定动作是：一位，做两臂屈肘扶头动作；二位，做两臂肩侧屈；三位，做两臂胸前交叉；四位，做两手叉腰。教师随意报出一至四之间的数字，看谁做得既快又好。 |

**表2 认知训练计划表**

| **训练时间** | **训练内容** | **举例/时间** | **详细步骤** | **图示** |
| --- | --- | --- | --- | --- |
| 1-4 week | 注意力，思考能力 | 辨别箭头移动方向游戏  (15min) | 测试开始后，屏幕中央将会序列呈现上下左右四种方向的箭头，箭头又分为粗细两种。每出现一个箭头，请你判断其粗细，粗箭头请按 D 键，细箭头请按 K 键。当一个序列呈现完毕，请你按顺序回忆刚才的箭头方向。 | 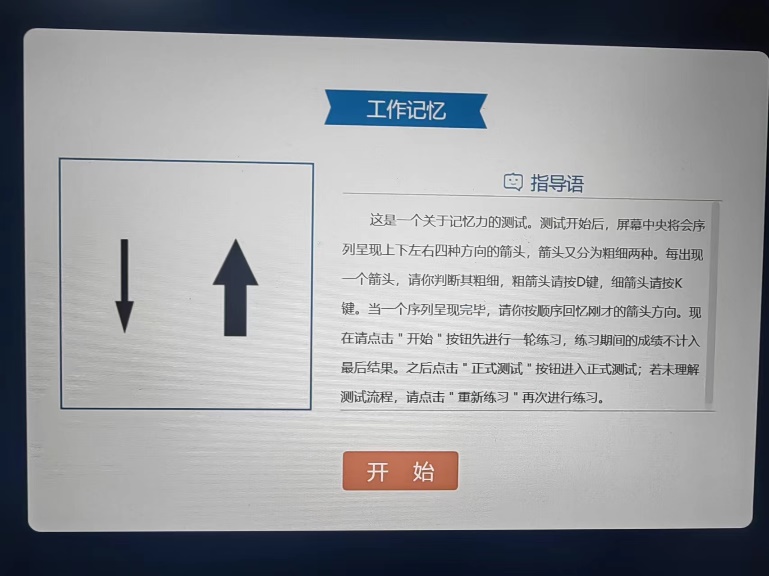 |
|  |  | 河内塔  (15min) | 测试开始后，屏幕将呈现 A 、 B 、 C 三个柱子， A 柱上放着几个圆盘，小的在上面，大的在下面。请你将 A 柱上的圆盘移到 C 柱上，条件是：每次只能移动任何一个柱子上的一个圆盘，但大的圆盘不可以放在小的圆盘上面，步数越少越好，时间越快越好。 | 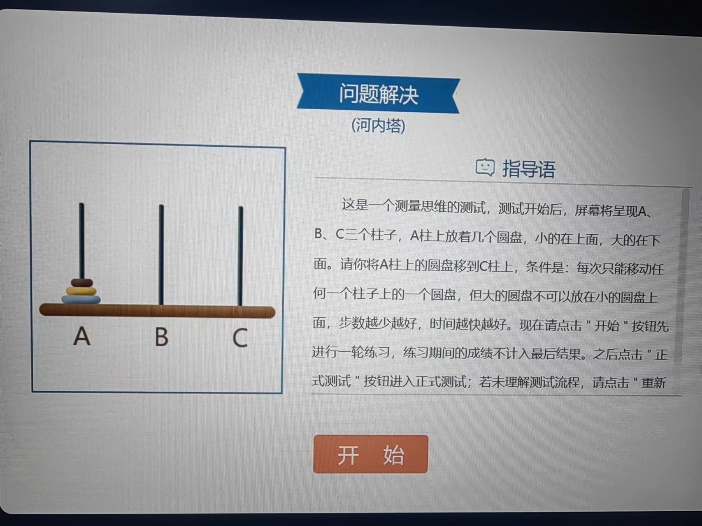 |
|  |  | 注意力集中  (15-20min) | 测试开始后，屏幕上会出现一个字符矩阵，请你在矩阵中从左到右，从上到下逐行按顺序检查矩阵，只要找到目标字符，就用鼠标左键点击进行标记，直到把此矩阵检查完，点击"下一页"继续检查。漏点和误点都被记录错误 | 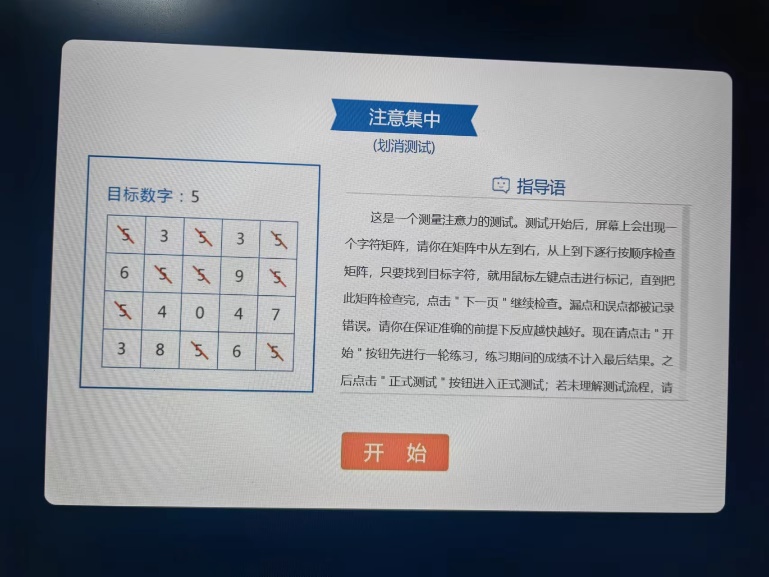 |
|  |  | 注意力瞬脱  (15min) | 测试开始后，屏幕中央会序列闪现多个字母，请记住红色的字母，并注意在红色字母出现后有没有字母 X 出现，然后回答下面的问题。 | 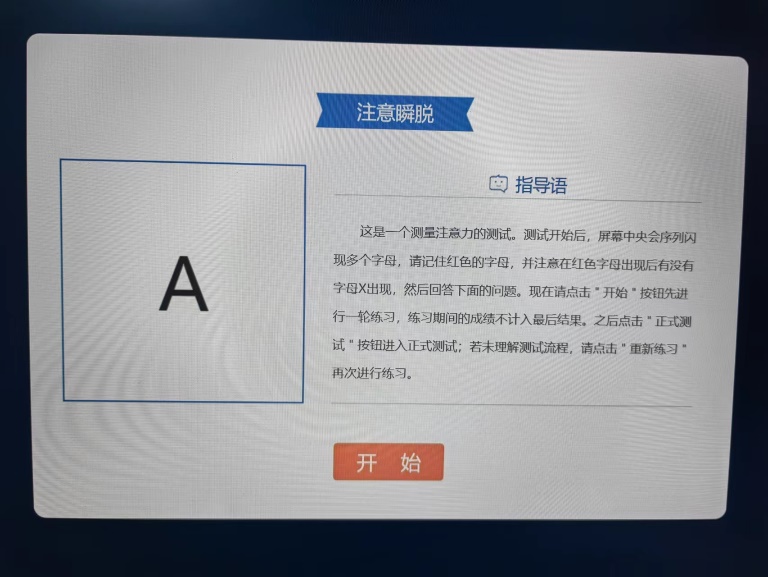 |
| 4-8 week | 反应时，感知训练 | 红圆和蓝圆  (15min) | 测试开始后，屏幕将显示红圆、蓝圆及"+"，你将完成两个任务，任务一为根据位置按键：目标出现在＋左边你要按左方向键，目标出现在＋右边你要按右方向键，忽略颜色。任务二为根据颜色按键：如果出现红圆你要按左方向键，出现蓝圆你要按右方向键，忽略位置。在保证正确的情况下反应越快越好。 | 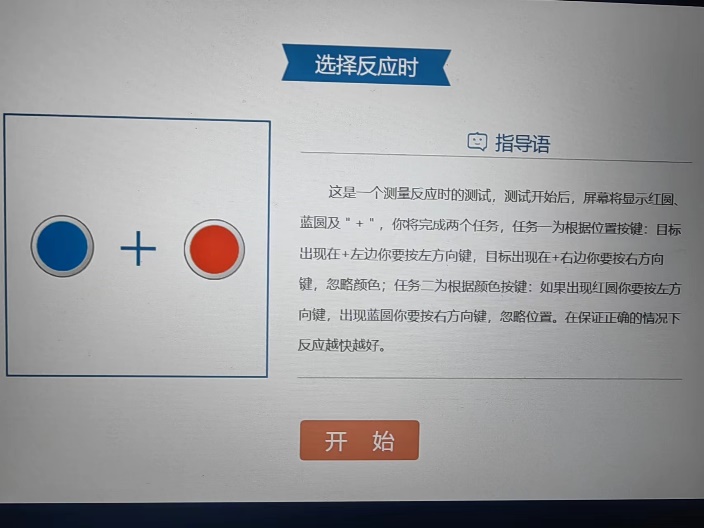 |
|  |  | 感知时间  (15min) | 屏幕会呈现一张图片，过段时间它会消失，请注意看它出现了多久。等图片消失后，按空格键，这时图片就又会出现，直到你觉得它呈现的时间和你刚才看到的一样长时，按下空格键。 | 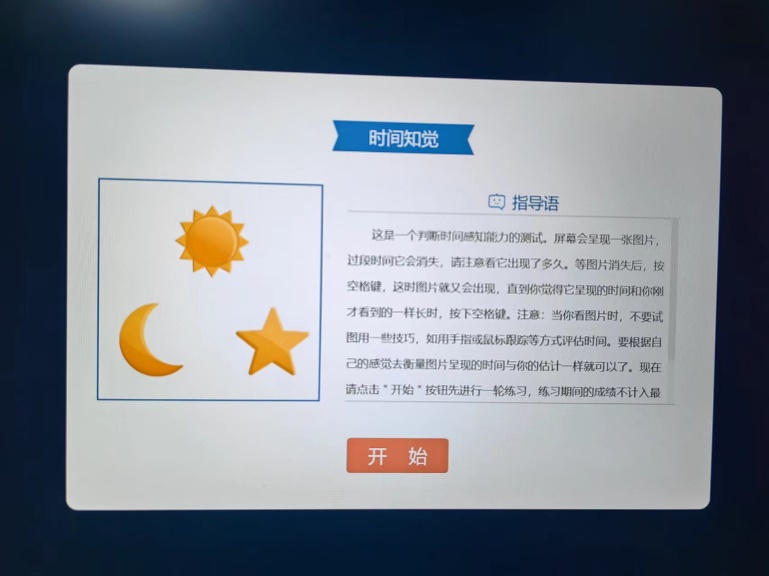 |
|  |  | 蓝色变绿色 | 点击电脑屏幕中间的矩形，等待颜色变为绿色后再次点击。 | 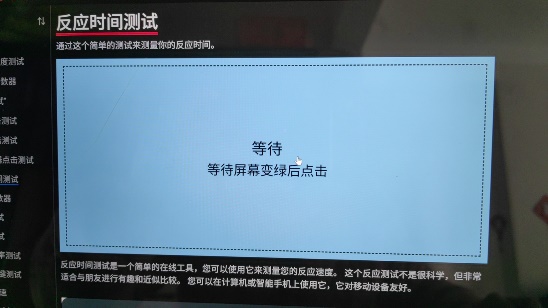 |
|  |  | 左-右  (10-15min) | 测试开始后，屏幕中央会出现一个中心点，之后会在其上方或下方出现一组箭头，请识别这组箭头中处于中间位置的箭头指向，并按下键盘上相应的方向键。 | 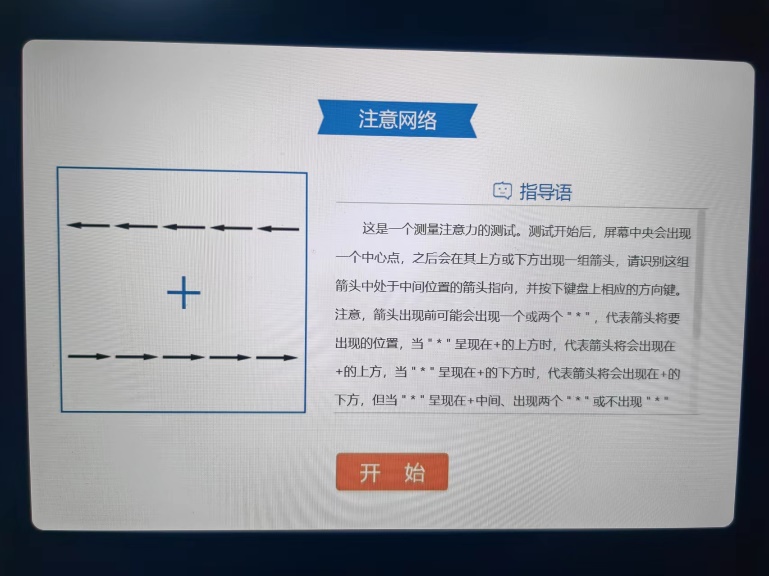 |
| 8-12 week | 记忆，认知灵活性 | 瞬时记忆  (15min) | 测试开始后，屏幕中央将呈现一组字符矩阵，你要尽可能地记住它们，很快会有一行内容消  失，请你回忆并将消失的内容输入到记录窗口中，并按空格键进入下一组矩阵。 | 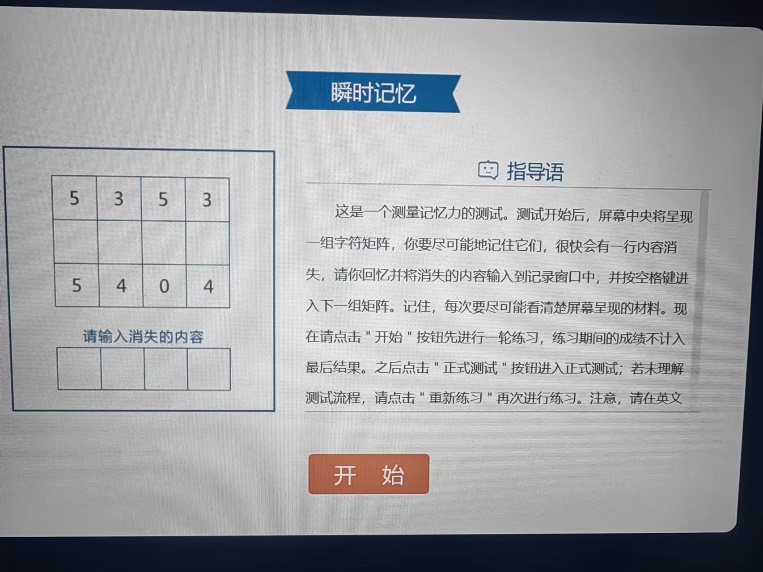 |
|  |  | 重复数字和字母  (15min) | 测试开始后，屏幕中央将呈现一串字符，你要尽可能地记住它们及其呈现顺序。很快它们消失后，屏幕上会呈现一个小游戏，游戏过后，请你将之前呈现的材料按照顺序回忆出来，并写在记录框中。 | 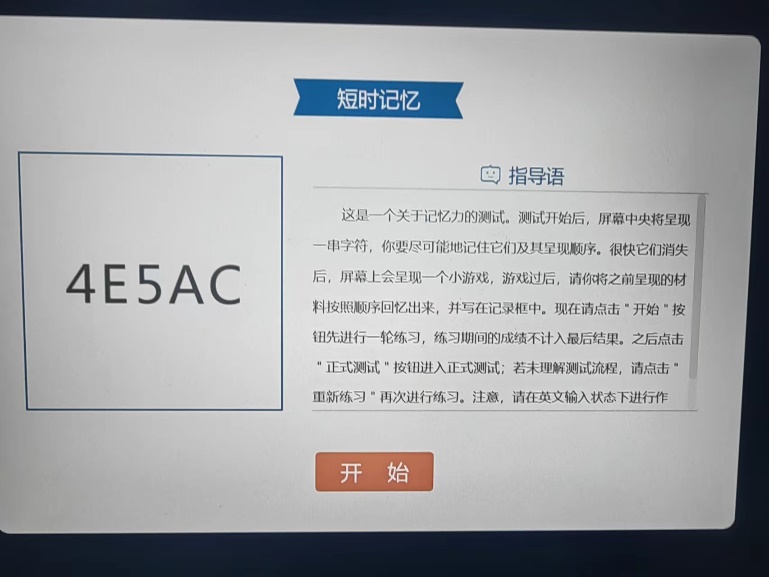 |
|  |  | 记忆分配  (15min) | 在下面的测试中，你将会看到红、黄、蓝一种圆及"上""下。"左""右"四个汉字。根据不同的条件，你的左右手将分别或配合完成一个任务：任务一是左手识别不同颜色的圆；任务二为右手识别代表方向的汉字，在任务二中，双手需要根据情况，分别对各自的目标做出反应。请在保持正确的情况下反应越快越好。另外，请在英文输入状态下进行作答。 | 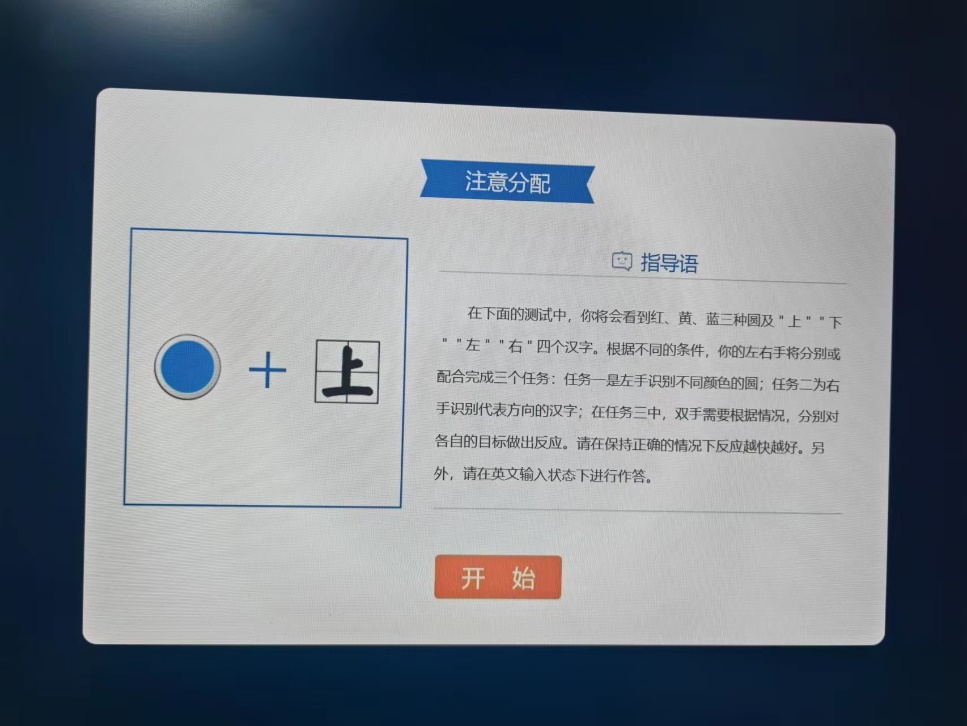 |
|  |  | 卡片分类  (15min) | 测试开始后，屏幕中将出现上下两部分卡片，卡片共有颜色、形状、数量三种分类方式，请判断下方的卡片与上方四个卡片中哪一个属于同一分类。连续判断正确十次后，将切换分类方式。 | 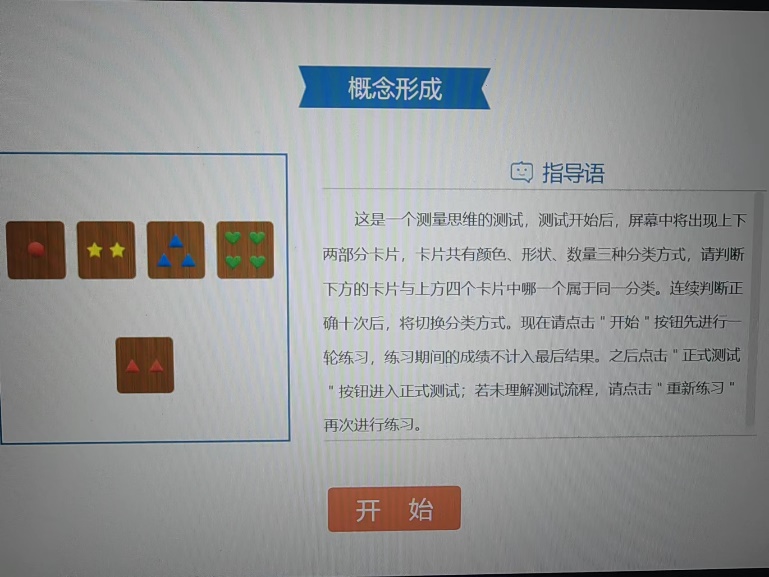 |
