# Supplementary material for: Comparing the effects of physical activity and cognitive training on cognitive performance, physical fitness, and mental health in 9- to 10-year-old children: a randomized clinical trial
Source: Front Psychol. 2025 Jun 26;16:1555451. doi: 10.3389/fpsyg.2025.1555451 (PMC12241119; doi:10.3389/fpsyg.2025.1555451)
Supplement: Supplementary file 3 [file Table_3.docx]

**Table 1 Physical activity schedule**

| **Training time** | **Training content** | **Example** | **Detailed steps** |
| --- | --- | --- | --- |
| 1-4 week | Balance, aerobic capacity | Drop the handkerchief  (15-20min) | All children sit in a circle. The first person to toss the handkerchief can either volunteer or be selected by the group. As everyone sings the nursery rhyme Diu Shou Juan ("The Handkerchief Toss") in unison, the tosser randomly places the handkerchief behind a player. The player who discovers the handkerchief behind them must immediately stand up and chase the tosser. If the tosser manages to return to and sit in their original spot before the song ends, the chased player is eliminated. |
|  |  | Relay-Run  (10-15min) | When the teacher gives the **"Ready"** command, the children assume the ready-to-run position. When they hear the **"Go"** command, they immediately start running the 50-meter distance. |
|  |  | Grass skiing  (15-20min) | Find a grassy slope outdoors and lay down a plastic sheet for children to slide down. Alternatively, have children sit in cardboard boxes or tubs to slide down. To add a sensory twist, spread a large plastic sheet on the slope, and when children slide down, adults can spray them with a hose, enhancing tactile stimulation and fun. |
|  |  | Balance beam  (10-15min) | Place the balance beam horizontally and have children first step over it using their hands for support. Once they master the movement, challenge them to cross without hand assistance. Gradually increase their crossing speed and introduce single-leg hops. Additionally, let children place both hands on the beam to challenge hand-supported jumps. |
| 4-8 week | Speed, and core stability | Obstacle Jump  (15-20min) | Children select a plastic pole and two identical shoe boxes to attempt leaping over obstacles at a specific height. Next, they can choose to practice leaping over hurdles at the original height or challenge themselves with higher obstacles. |
|  |  | Crab crawling  (10-15min) | Play the Pang Xie Ti Cao ("Crab Exercise") music while all children follow along in unison by mimicking the crab-like movements. |
|  |  | throw the bean bag  (10-15min) | Have the child begin by throwing smaller paper balls as far as possible, then switch to slightly larger ones, followed by sandbags. Repeat the exercise to reinforce throwing skills. |
| 8-12 week | Reactivity and flexibility | Go/No go  (15-20min) | Participants must decide whether to run based on audio cues: refrain from running upon hearing the "Run" command, but immediately start running when the "Don't Run" instruction is given. Concurrently, when viewing images, they must determine whether to press a button based on whether the displayed word ends with "R" (press) or "N" (no press). |
|  |  | Multi-directional movement  (15-20min) | Linear-direction movement: Encompasses rapid movements in four orientations — forward, backward, left, right. Curvilinear-direction movement: Involves swift motion along three trajectory types — arc-shaped, circular, oval paths. |
|  |  | Turn Left, turn right (10-15min) | Children raise both hands to ear level, slightly bend their knees, and crab-walk sideways continuously left and right. Alternatively, they may gently lower their arms, tilt their head sideways, and step forward, left, or right. Another variation involves extending arms straight forward or out to the sides while stepping in multiple directions. |
|  |  | Do the action as instructed by the command  (10-15min) | Before the game starts, define four positions: **Position 1**: Bend elbows and place hands on head; **Position 2**: Bend arms laterally at shoulder level; **Position 3**: Cross arms over chest; **Position 4**: Place hands on hips. The teacher randomly calls out numbers 1–4, and children must assume the corresponding pose as quickly and accurately as possible. |

**Table 2 Cognitive training schedule**

| **Training time** | **Training content** | **Example** | **Detailed steps** | **Graphical representation** |
| --- | --- | --- | --- | --- |
| 1-4 week | Attention, thinking ability | Discerning the direction of arrow movement (15min) | After the test begins, arrows pointing up, down, left, or right will be presented sequentially at the center of the screen, each appearing in either thick or thin line weight. When each arrow appears: Press D for thick arrows, K for thin arrows. After a sequence is completed: Recall the arrow directions in their original presentation order. | 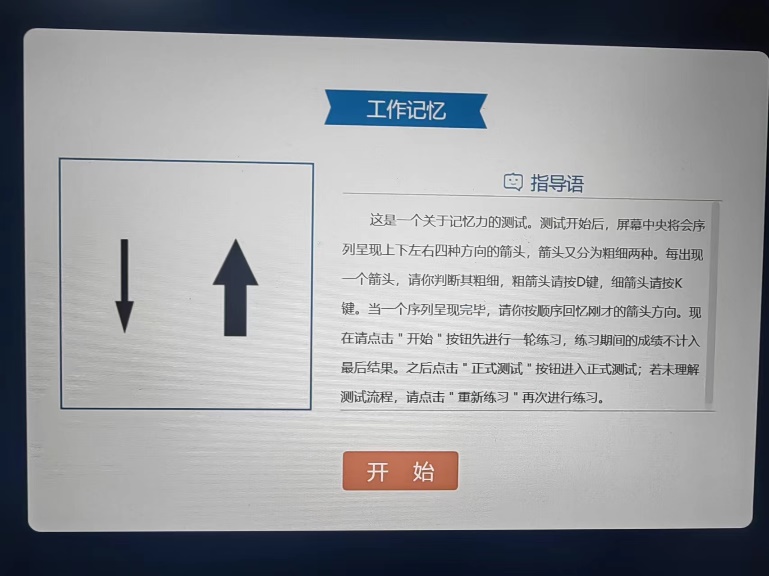 |
|  |  | Hanoi tower  (15min) | After the test begins, three vertical pegs labeled A, B, and C will appear on the screen. Peg A initially holds several disks arranged with smaller disks stacked atop larger ones. Your task is to transfer all disks from Peg A to Peg C under the following rules: Move only one disk at a time from any peg. Never place a larger disk on top of a smaller one. The goal is to achieve the transfer in the minimum number of moves and shortest time possible. | 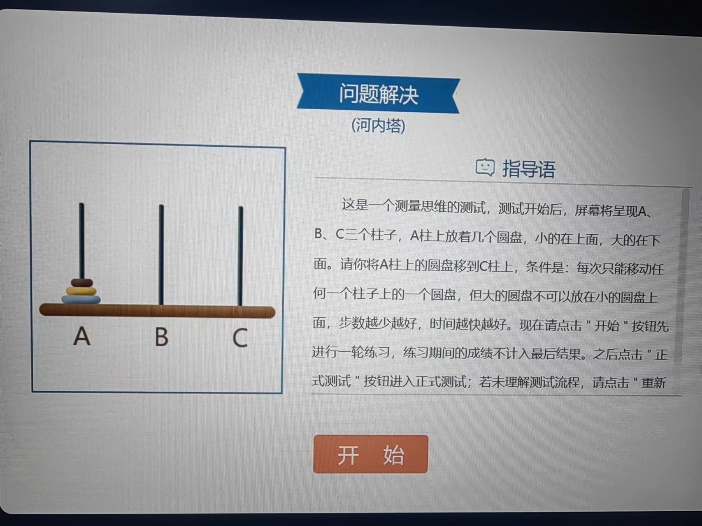 |
|  |  | Attention concentrativeness  (15-20min) | After the test begins, a matrix of characters will appear on the screen. You must systematically scan the matrix from left-to-right and top-to-bottom in sequential row order. Whenever you identify a target character, left-click to mark it. Continue this process until the entire matrix is scanned, then click "Next Page" to proceed. Both missed targets (unmarked valid characters) and false positives (incorrectly marked ones) are recorded as errors. | 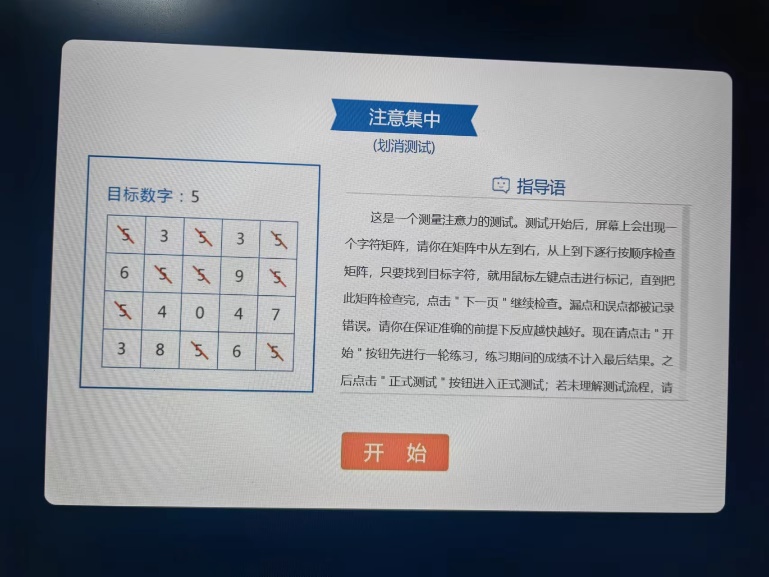 |
|  |  | Attentional blink (15min) | After the test begins, multiple letters will be sequentially flashed at the center of the screen. Participants must memorize all red-colored letters and monitor whether the letter X appears immediately after any red-colored letter. Subsequently, they will answer follow-up questions based on these observations. | 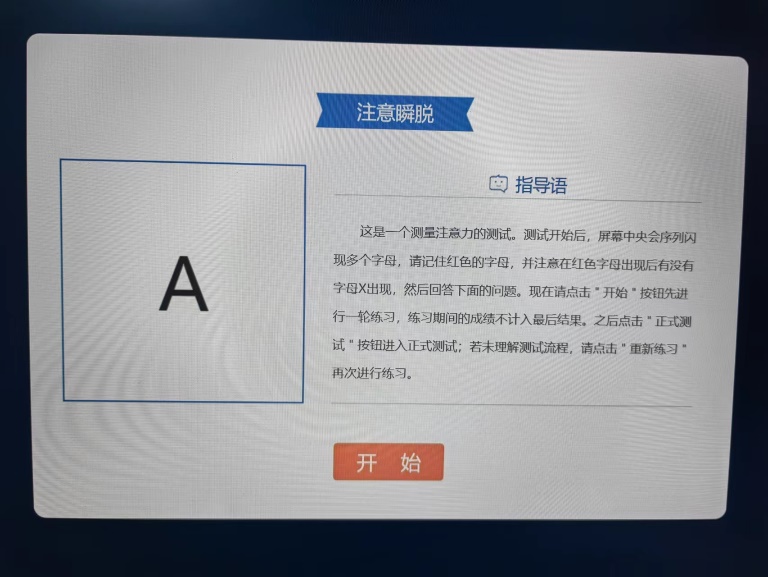 |
| 4-8 week | Reactive time, perception | Red and Blue  (15min) | After the test begins, the screen will display red circles, blue circles, and a fixation cross (+). You will complete two distinct tasks:Task 1 (Spatial Response): Press the left arrow key if a target appears to the left of the +, Press the right arrow key if it appears to the right of the +. Ignore all color information in this task. Task 2 (Chromatic Response): Press the left arrow key for red circles, Press the right arrow key for blue circles. Ignore all spatial locations in this task. | 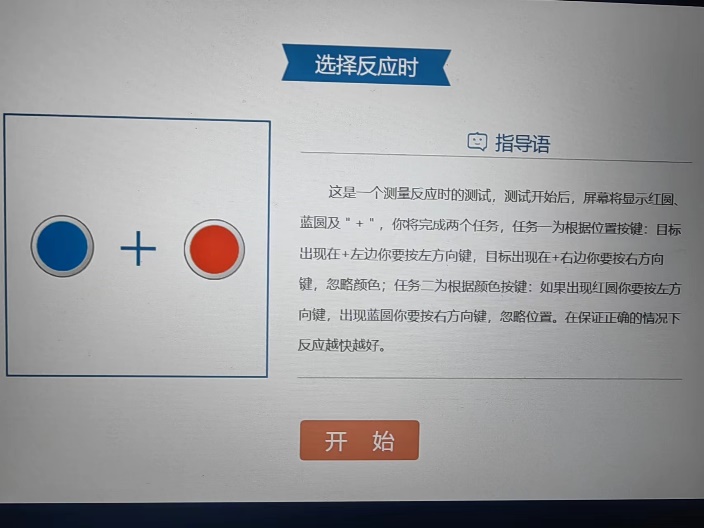 |
|  |  | Perception time (15min) | A picture will be displayed on the screen and disappear after a variable duration. Carefully observe how long it remains visible. Once it vanishes, press the spacebar to make it reappear. Continue holding the spacebar until the re-displayed duration matches your perception of the initial presentation time, then release the key. | 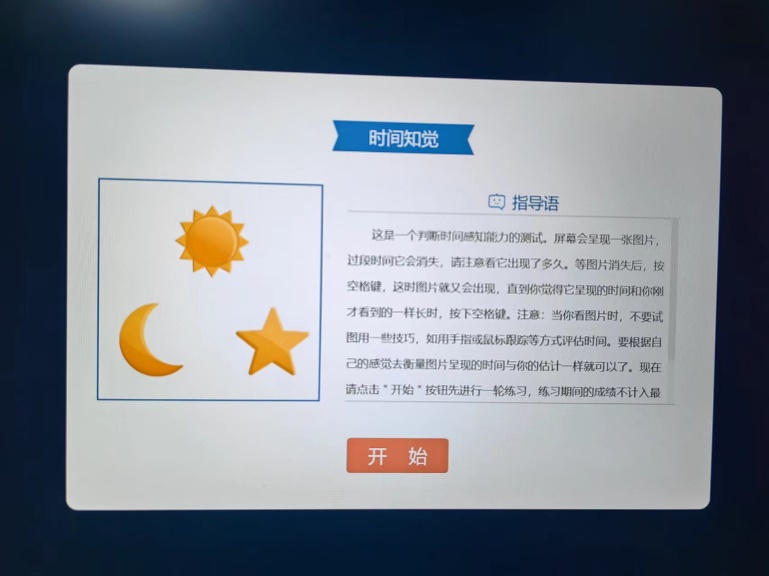 |
|  |  | Red and Blue | Click the rectangle at the center of the screen, wait until it turns green, then click it again. | 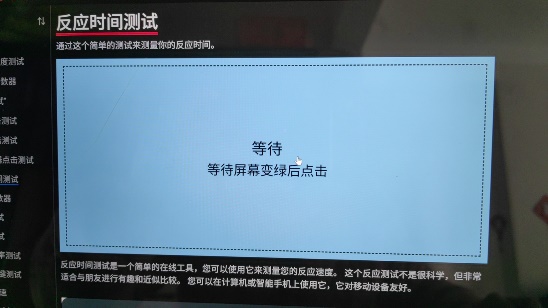 |
|  |  | Left-Right  (10-15min) | After the test begins, a central fixation point will appear on the screen. Subsequently, an array of arrows will be displayed either above or below this point. Identify the direction of the middle arrow in the array and press the corresponding direction key (← or →) on your keyboard. | 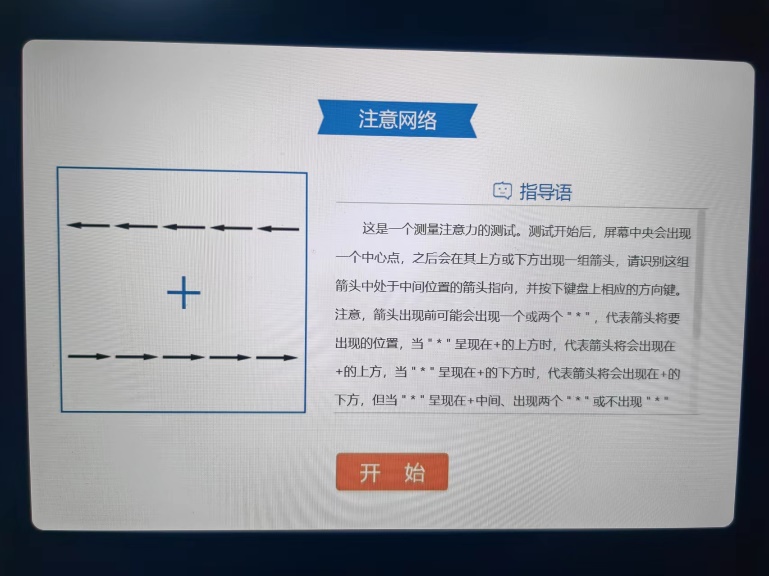 |
| 8-12 week | Memory, cognitive flexibility | instantaneous memory  (15min) | After the test begins, a matrix of characters will be displayed at the center of the screen. Memorize the characters to the best of your ability. Shortly after, one row will disappear. Recall the missing content and type it into the response window, then press the spacebar to advance to the next matrix set. | 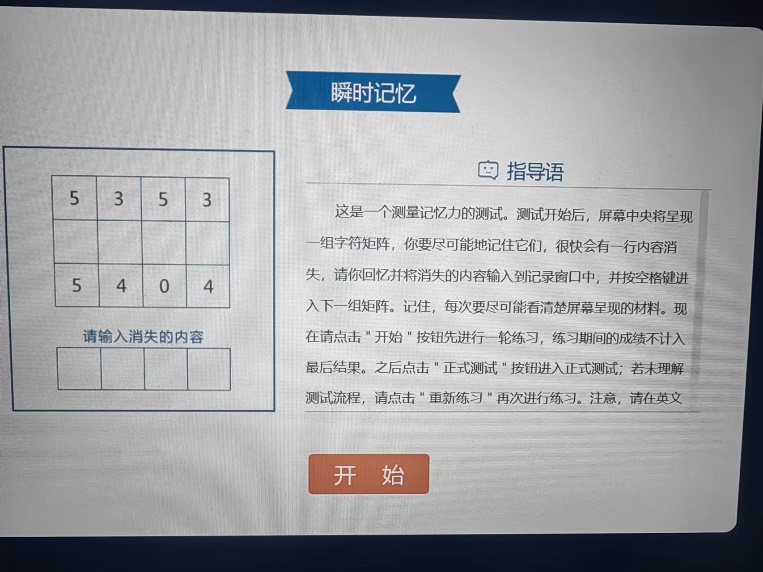 |
|  |  | Repeated numbers and letters (15min) | After the test begins, a sequence of characters will be displayed at the center of the screen. Memorize both the characters and their presentation order to the best of your ability. Shortly after they vanish, a brief distractor task (e.g., a mini-game) will appear. Once the task concludes, recall the original sequence in the correct order and input your responses into the designated recording box. | 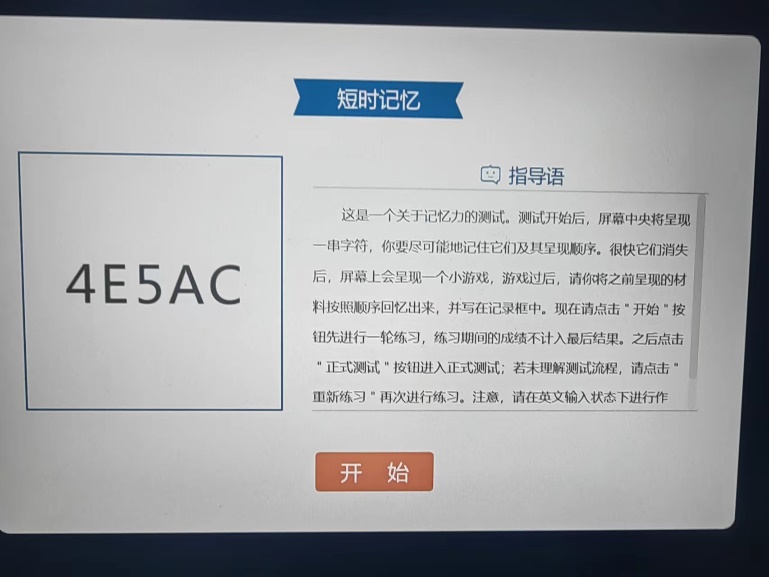 |
|  |  | Memory allocation  (15min) | In the following test, you will see three colored circles (red, yellow, blue) and four directional Chinese characters: 上 (up), 下 (down), 左 (left), 右 (right). Depending on the condition, your left and right hands will perform either independent or coordinated tasks: Task 1 (Color Recognition): Left hand: Identify the color of the circles (red/yellow/blue) using designated keys. Ignore all directional characters during this task. Task 2 (Direction Discrimination): Right hand: Identify the directional characters (上/下/左/右) using corresponding arrow keys. Both hands must independently respond to their respective targets based on contextual cues. Respond as quickly as possible while maintaining accuracy. Ensure your keyboard is in English input mode to avoid input conflicts. | 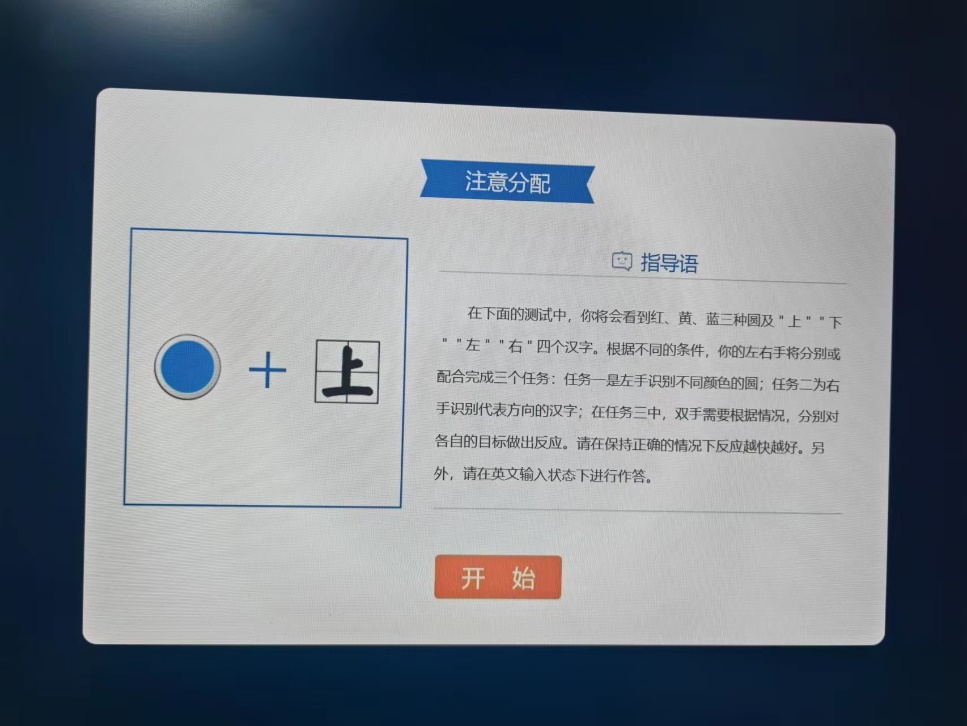 |
|  |  | Card-sorting  (15min) | After the test begins, the screen will display two sections of cards:   - Upper section: Four cards categorized by color, shape, or quantity. - Lower section: One target card.   Your task is to determine which upper card shares the same classification (color/shape/quantity) as the lower card. After 10 consecutive correct judgments, the system will automatically switch the classification criterion. | 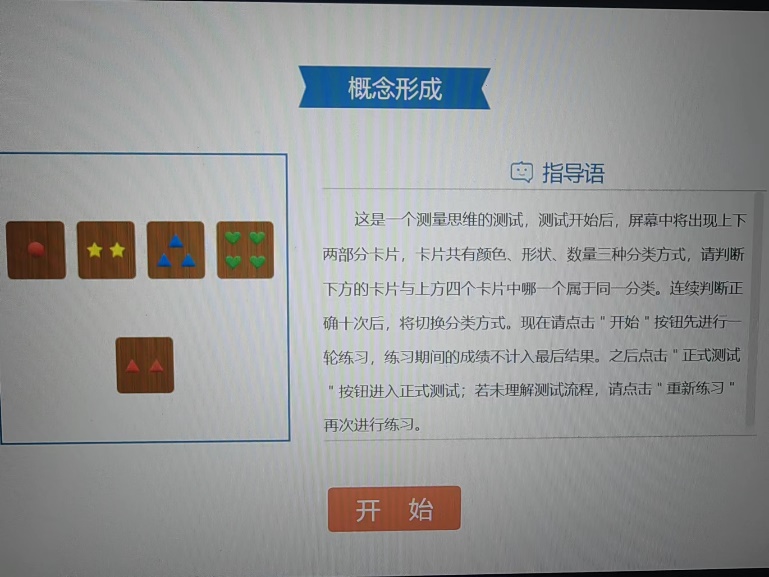 |
